# Reinforcing Mechanism of Reduced Graphene Oxide on Flexural Strength of Geopolymers: A Synergetic Analysis of Hydration and Chemical Composition

**DOI:** 10.3390/nano9121723

**Published:** 2019-12-03

**Authors:** Wu-Jian Long, Tao-Hua Ye, Qi-Ling Luo, Yaocheng Wang, Liu Mei

**Affiliations:** Guangdong Provincial Key Laboratory of Durability for Marine Civil Engineering, Shenzhen Durability Center for Civil Engineering, College of Civil and Transportation Engineering, Shenzhen University, Shenzhen 518060, China; longwj@szu.edu.cn (W.-J.L.); yetaohua2017@email.szu.edu.cn (T.-H.Y.); luoqiling@szu.edu.cn (Q.-L.L.); wangyc@szu.edu.cn (Y.W.)

**Keywords:** reduced graphene oxide, reduction degree, geopolymer, flexural strength, hydration, chemical composition, reinforcing mechanism

## Abstract

With the development of nanotechnology, reduced graphene oxide (rGO) has been used to improve the flexural strength of geopolymers. However, the reinforcing mechanism of rGO nanosheets on the flexural strength of geopolymers remains unclear. Here, this reinforcing mechanism was investigated from the perspectives of hydration and chemical composition. The effect of the reduction degree on rGO-reinforced geopolymers was also studied using isothermal calorimetry (IC), X-ray diffraction (XRD), and nuclear magnetic resonance (NMR) tests. Results show that the hydration degree and flexural strength of geopolymers effectively increase due to rGO addition. After alkali reduction at a temperature of 60 °C, rGO nanosheets have maximum reinforcement on the flexural strength of geopolymers with an increment of 51.2%. It is attributed to the promotion of slag hydration, as well as the simultaneous formation of calcium silicate hydrate with low Ca/Si ratio (C-S-H(I)) and calcium aluminosilicate hydrate (C-A-S-H) phases due to the inhibiting effect of rGO nanosheets on Al substitution on the end-of-chain silicates of C-S-H and C-A-S-H gels. In addition, different reduction degrees have almost no effect on the chemical composition of rGO-reinforced geopolymers, while excessive reduction impairs the improving effect of rGO nanosheets on the hydration process and flexural strength of geopolymers due to significant structural defects.

## 1. Introduction

Geopolymers are an interesting class of inorganic materials and are synthesized through the chemical reaction between a highly alkaline solution and aluminosilicate precursors. Generally, geopolymers have much less of an environmental footprint than ordinary Portland cement (OPC), since their precursors are mainly made from industrial by-products such as ground granulated blast furnace slag. Previous studies [1,2,3] have demonstrated that geopolymers exhibit high early compressive strength, excellent thermal resistance, and superior durability. Owing to their greater environmental benefits and comparable performances with OPC, geopolymers are regarded as one of the most promising binders for construction. However, because of their quasi-brittle nature, low flexural strength of geopolymers largely hinders their practical application [4].

With the development of two-dimensional (2D) nanotechnology, the feasibility of 2D nanomaterials to enhance the flexural strength of geopolymers has been investigated. Graphene, which is a 2D planar sheet of single-atom thickness [5,6] with an excellent specific surface area of 2630 m^2^/g [7], possesses an ultrahigh Young’s modulus of 1 TPa [8], an ultimate strength of 130 GPa [8], an exceptional thermal conductivity of 5000 W/(m·K) [9], and an electrical conductivity of 2000 S/cm [10]. Currently, graphene derivatives mainly exist in two forms: Graphene oxide (GO) and reduced graphene oxide (rGO). In particular, rGO nanosheets share a few characteristics with GO nanosheets [11] and are generally prepared through the heat or alkali reduction of GO nanosheets. It has been reported that rGO nanosheets have a few residual oxygen-containing functional groups such as epoxy (COC), carbonyl (CO), hydroxyl (OH), and carboxyl (COOH) [12]. These functional groups can assist rGO nanosheets to effectively disperse in water owing to electrostatic stabilization, and uniformly disperse in the geopolymer matrix as well [13]. Given these advantages, Saafi et al. [14] introduced rGO nanosheets into geopolymers and found that 0.35 wt % rGO addition can improve the flexural strength by 134% due to the formation of a pore-filled morphology. Shu et al. [13] also reported that 0.3 wt % rGO addition can effectively enhance the flexural strength of geopolymers owing to the strong interface bonding, the cracking deflection, and propagation. Up to date, rGO nanosheets have been proven to mitigate the quasi-brittleness of geopolymers. However, the reinforcing mechanism of rGO nanosheets on the flexural strength of geopolymers remains unclear. In addition, the effects of rGO nanosheets on the hydration process and chemical composition are also unclear.

Reduction degree, i.e., the number of oxygen-containing groups, is an important index to evaluate the properties of rGO nanosheets. Shu et al. [15] investigated the effects of external heating on the reduction degree and flexural strength of rGO-reinforced geopolymers. They indicated that rGO nanosheets can further undergo reduction in the hardened geopolymers during heat treatment, and 1 wt % rGO addition can increase the flexural strength of the hardened geopolymers by 120% after heating above 1000 °C for 30 min. However, it should be noted that external heating can lead to other issues in the hardened geopolymers such as the decomposition of hydration products, the coarsening of the pore structures, and the formation of cracks [16,17]. Thus, the sole effect of the reduction degree of rGO nanosheets on the flexural strength of geopolymers needs to be further studied. In addition, the effects of the reduction degree on the hydration process and chemical composition are still unclear.

Based on that mentioned above, rGO nanosheets with different reduction degrees were synthesized using a 10 mol/L NaOH solution at different temperatures in this study. The contribution is aimed at: (1) Investigating the effects of the reduction degree of rGO nanosheets on the hydration process, chemical composition, and flexural strength of geopolymers using isothermal calorimetry (IC), X-ray diffraction (XRD), and nuclear magnetic resonance (NMR) tests; (2) illustrating the reinforcing mechanism of rGO nanosheets on the flexural strength of geopolymers through a synergistic analysis of the hydration process and chemical composition. The findings of this work can provide not only insights into the performance of rGO-reinforced geopolymers, but also a guide for their oriented design.

## 2. Materials and Methods

### 2.1. Raw Materials

**Figure 1:**
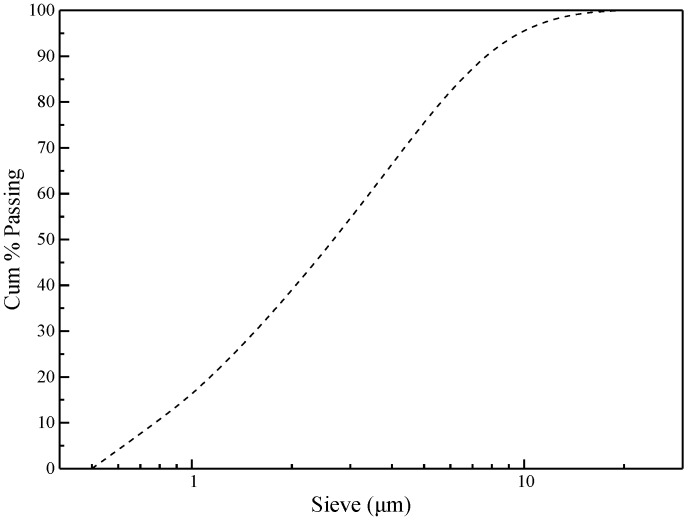
Particle size distribution of slag.

Ground granulated blast furnace slag used in this study conformed to the requirements of the Chinese Standard GB/T 18,046 [18]. Figure 1 shows the corresponding particle size distribution of the slag. Table 1 lists the chemical compositions and physical properties of the slag. A mixture of solid sodium hydroxide (NaOH, 96% purity, Guanghua Technology Co., Ltd., Shantou, Guangdong, China) and waterglass (Na_2_SiO_3_ with 8.8 wt % Na_2_O, 27.8 wt % SiO_2_, and 63.4 wt % H_2_O, Shengjing Ceramic Materials Co., Ltd., Zhaoqing, Guangdong, China) was used as the alkaline activator in this study. Graphite oxide powder from Sixth Element Materials Technology Co., Ltd. (Changzhou, Jiangsu, China) was used to prepare the GO solution in the experiment. The properties of the powder are listed in Table 2.

### 2.2. Test Methods

#### 2.2.1. Preparation of GO Solution

To prepare the GO solution, a specific amount of graphite oxide powder was magnetically stirred with deionized water for 30 min. The resulting aqueous suspension with a concentration of 16.7 g/L was treated for 2 h using an ultrasonic homogenizer with 400 W and 25 Hz. To avoid suspension overheating by ultrasonication, the treatment was conducted in cycles (an operation period of 2 s and an interval of 4 s). After the ultrasonication, the obtained GO solution was used to prepare rGO solution and rGO-reinforced geopolymers [19,20].

#### 2.2.2. Sample Preparation

**Figure 2:**
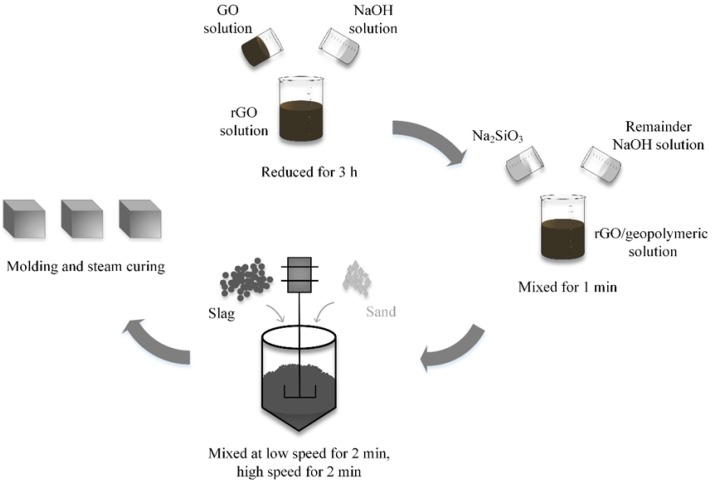
Mixing procedures of reduced graphene oxide (rGO)–geopolymer pastes and mortars. Note: Sand is only used for the preparation of the mortars.

The rGO–geopolymer pastes and mortars were prepared at a rGO-to-slag ratio of 0.003 and a water-to-slag ratio of 0.6. The alkali dosages (Na_2_O/slag) and silicate modulus (SiO_2_/Na_2_O) were 0.08 and 1.2, respectively. A sand-to-slag ratio of 3.0 was used for preparing the mortars. Figure 2 shows the mixing procedures of the pastes and mortars. First, the dispersed GO solution was divided into four parts. Each part was added to 100 g of NaOH solution with a concentration of 10 mol/L and mildly stirred for 10 min. To prepare rGO with different reduction degrees, four mixtures were cured for 3 h at temperatures of 25, 40, 60, and 80 °C. Subsequently, each stable and heterogeneous mixture was mixed with liquid materials (remainder NaOH solution + waterglass) for 1 min. Finally, the rGO/geopolymeric solution and solid materials (slag for pastes or slag + sand for mortars) were mixed at a rotation speed of 62 ± 5 rpm for 2 min, followed by mixed at a rotation speed of 125 ± 10 rpm for another 2 min. The obtained rGO–geopolymer composites were cast into two molds (dimensions: 40 × 40 × 160 mm^3^ and 15 × 15 × 15 mm^3^) and cured at a temperature of 20 ± 2 °C and a relative humidity > 95% for 24 h. According to Shi et al. [21], alkali-activated materials cured at 80 °C for 48 h exhibit the highest mechanical strength, since the alkali activation is almost complete. Thus, the demolded samples were steam cured in a chamber maintained at 80 °C for 48 h. The blank sample and the samples with the addition of rGO nanosheets reduced at 25, 40, 60, and 80 °C were denoted by M0, R1, R2, R3, and R4, respectively.

#### 2.2.3. Characterization of rGO Nanosheets

The rGO solutions with different reduction degrees were prepared using the same procedure and characterized using two experiments: (1) Raman scattering (532 nm, type inVia Reflex, Renishaw, London, UK) was used to reflect the structural defects in the rGO nanosheets and (2) transmission electron microscopy (TEM, type Talos 200F, FEI, Hillsboro, OR, USA) was applied to scan their morphologies.

#### 2.2.4. Heat of Hydration

After the rGO–geopolymer pastes were prepared, 8 g of the paste was used for hydration characterization. The normalized heat flow and cumulative heat of the pastes were measured for 150,000 s using an isothermal calorimeter with eight channels (type TAM air, TA instruments, New Castle, DE, USA). This instrument has a wide temperature measurement range from 5 to 90 °C, with the temperature being controlled within ±0.02 °C.

#### 2.2.5. X-Ray Diffraction (XRD) Test

The chemical compositions of the hardened pastes were determined by the XRD test, performed on a DX 2500 (Dandong Fangyuan Instrument, Liaoning, China). A Cu anode was used and the X-ray diffractometry was carried out at 40 kV and 40 mA. The samples were scanned in the range from 5° to 80° with a rate of 2°/step.

#### 2.2.6. Nuclear Magnetic Resonance (NMR) Test

Solid-state ^29^Si magic angle spinning (MAS) NMR spectra were collected at 119.2 MHz on a JNM-ECZ600R/M1 spectrometer (JEOL, Tokyo, Japan) using a spinning speed of 10.0 kHz and a probe for 3.2 mm zirconia rotors. For the ^29^Si MAS experiments, the pulse width, relaxation delay, and the number of scans were 0.1 µs, 20 s, and 1000, respectively. Solid-state ^27^Al MAS NMR spectra were collected at 156.4 MHz on the same instrument. The pulse width, relaxation delay, and the number of scans were 0.1 µs, 5 s, and 32, respectively. The ^29^Si and ^27^Al chemical shifts were referenced to the external samples of tetramethylsilane (TMS) and AlCl_3_·6H_2_O solution with a concentration of 1.0 mol/L, respectively.

#### 2.2.7. Flexural Strength Test

The flexural strength of the hardened mortars from each group was conducted on a computerized universal testing machine (YHZ-300, Luda Mechanical Instrument Co., Ltd., Shaoxing, Zhejiang, China). The flexural strength test was operated at a loading rate of 500 N/s, conforming to the Chinese standard GB/T 17671 [22]. Finally, the average of three tested values of each group was calculated as the flexural strength for such group.

## 3. Results and Discussion

### 3.1. Characterization of rGO Nanosheets

**Figure 3:**
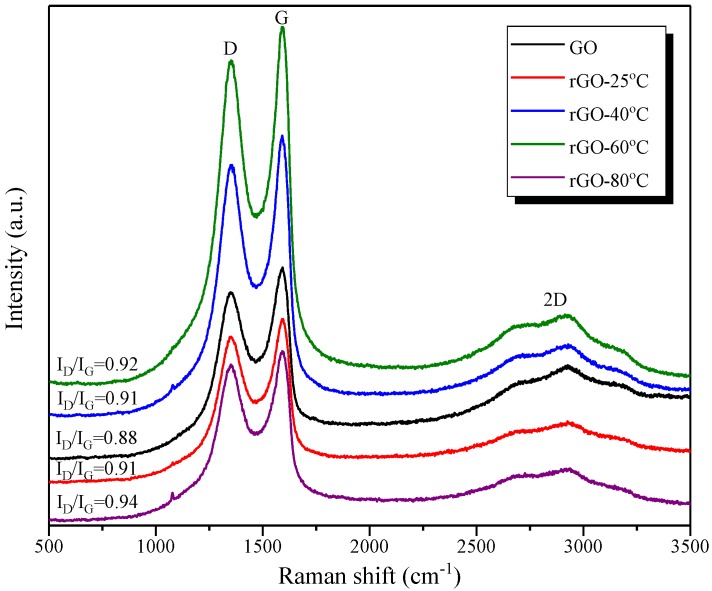
Raman spectra of graphene oxide (GO; black line) and rGO obtained after reduction under the 10 mol/L NaOH solution at 25 °C (red line), 40 °C (blue line), 60 °C (green line), and 80 °C (purple line).

In this work, the effects of the reduction degree on the hydration degree, chemical composition, and flexural strength of rGO-reinforced geopolymers were investigated. To obtain rGO nanosheets with different reduction degrees, GO nanosheets were reduced in a 10 mol/L NaOH solution at temperatures of 25, 40, 60, and 80 °C, respectively. Figure 3 shows the Raman spectra of the GO and rGO nanosheets obtained after the reduction, where two dominant peaks can be clearly observed. The first band at 1620 cm^−1^, G-band, is formed by the stretching of the C–C bond in the graphite mode. The second band at 1380 cm^−1^, D-band, derives from the diamondoid mode. The third band at 2900 cm^−1^, 2D band, was the second order of the D peaks. In particular, the ratio of the intensity of the D-band (I_D_) to that of the G-band (I_G_) is proportional to the structural defects in graphene, i.e., the higher the value of ID/IG, the greater the structural defects [23,24]. As shown in Figure 3, the value of I_D_/I_G_ of the sample increases from 0.88 to 0.94 with elevated temperatures, indicating an increasing number of structural defects in rGO structures after reduction.

**Figure 4:**
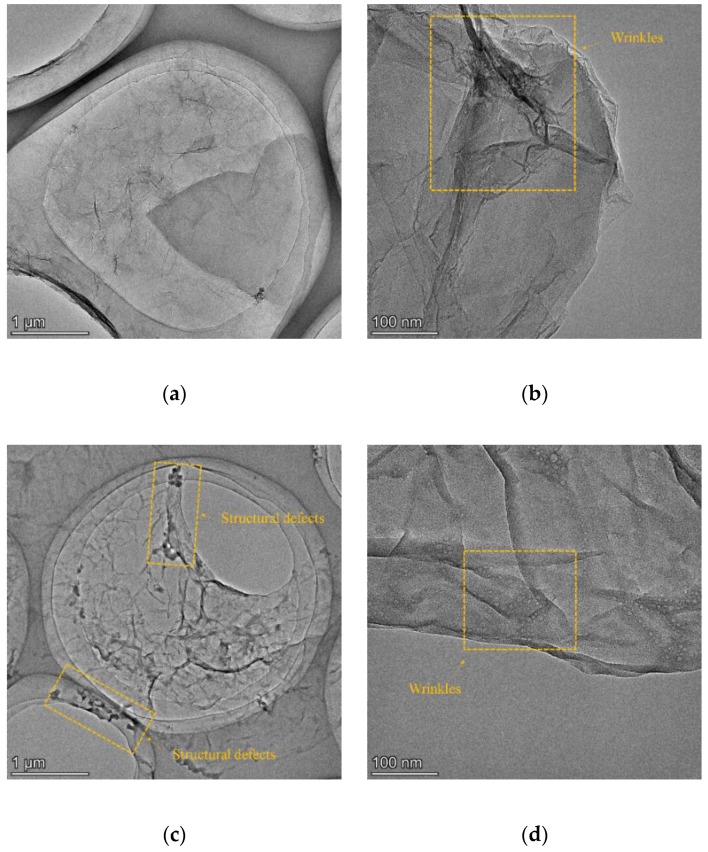

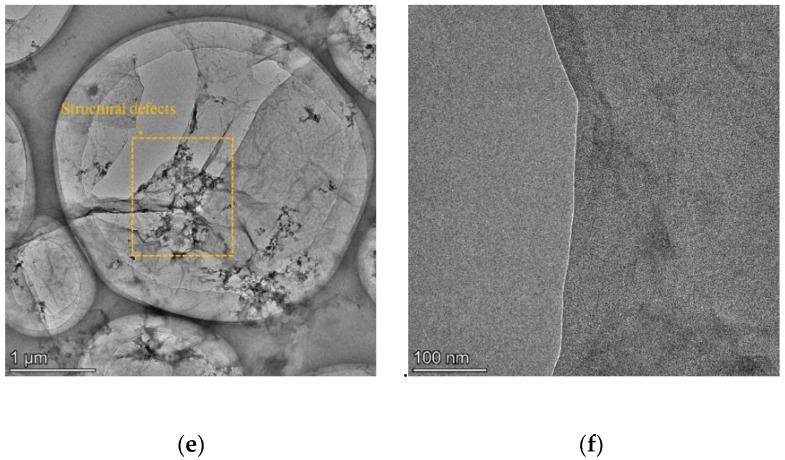
TEM images of GO (**a**,**b**), rGO after reduction at 40 °C (**c**,**d**), and rGO after reduction at 80 °C (**e**,**f**).

Figure 4 shows the TEM images of the GO and rGO nanosheets after reduction at temperatures of 40 and 80 °C, which confirms that the graphene-based materials have an irregular sheet-like structure. As shown in Figure 4a,c,e, the structural defects in rGO nanosheets increase with elevated temperatures, which is in line with the Raman results. According to Long et al. [25,26], the wrinkled and folded areas on the GO surface derive from the intercalation of the abundant oxygen-containing groups. Thus, the number of oxygen-containing groups on rGO nanosheet decreases with elevated temperatures due to alkali reduction, as shown in Figure 4b,d,f. It can be demonstrated that GO nanosheets can be effectively reduced in the 10 mol/L NaOH solution and that a higher reduction degree can be achieved at a higher treatment temperature.

### 3.2. Hydration Process

**Figure 5:**
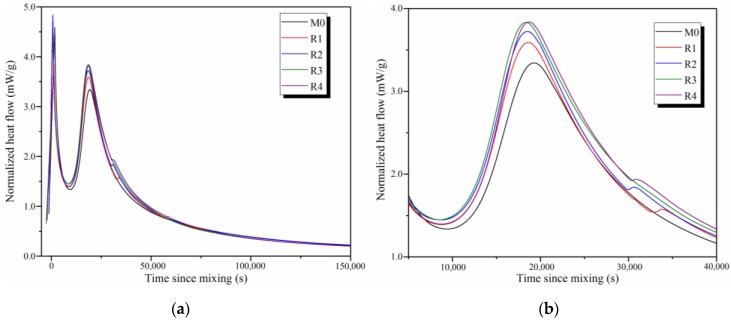
Normalized heat flow of M0, R1, R2, R3, and R4: (**a**) the overall view and (**b**) the enlarged partial view.

Figure 5a shows the overall view of the normalized heat flow of M0, R1, R2, R3, and R4. Results show that the hydration process of the slag-based geopolymers is similar to that of OPC and can be divided into five stages: The initial, induction, acceleration, deceleration, and steady-state stages. The first peak appears immediately after the mixing was complete, corresponding to the wetting and initial dissolution of the slag particles [27]. Most of the decomposition reaction happens in this period. Following the first peak, a short dormant period can be detected as the induction stage, providing the necessary time for the increase of the concentrations of one or two valence metal ions (Ca, Na, Al, etc.) produced from the dissolution of the slag in the alkali solution [28]. This period can be also explained by the delayed nucleation theory [29]. Due to the continuous dissolution of the slag, the concentrations of these ions in the pore solution exceed the saturation concentrations of calcium silicate hydrate (C-S-H) and calcium aluminosilicate hydrate (C-A-S-H), thus leading to the polymerization and condensation of C-S-H and C-A-S-H phases [30]. As a result, the second peak represents the acceleration stage of the hydration process. It should be noted that the second peak in this study occurs approximately 20,000 s after mixing, whereas the second peak for waterglass-activated geopolymers in other studies was detected after approximately 100,000 s or even later [31,32,33]. According to Zhou et al. [30], this difference can be attributed to the different compositions of the alkali activators used. Finally, the hydration process falls in the deceleration and steady-state stages due to the infilling and accretion of the hydration products formed [31].

**Figure 6:**
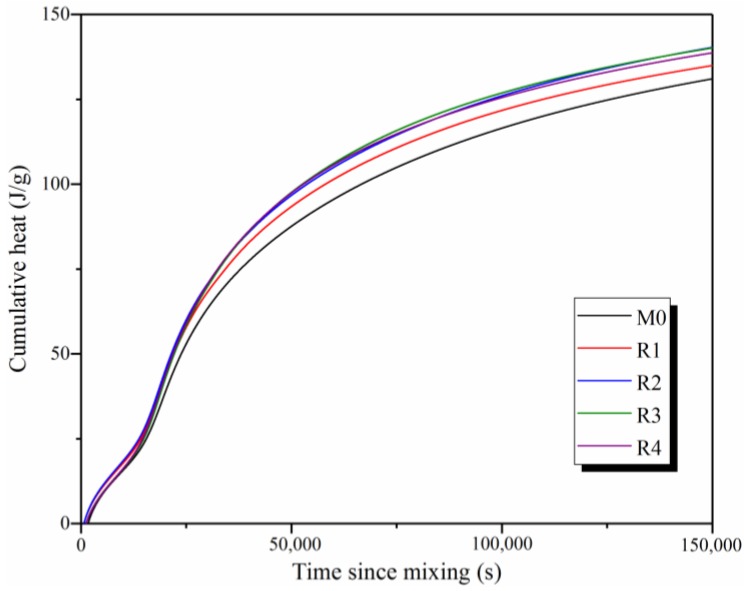
Cumulative heat of M0, R1, R2, R3, and R4.

To investigate the effects of rGO addition and its reduction degree on the hydration process of slag-based geopolymers, Figure 5b shows the enlarged partial view of the normalized heat flow of the samples. Results show that the second peak for R3 (18,200 s) appears the earliest, followed by R2 (18,500 s), R1 (18,600 s), R4 (18,700 s), and M0 (19,200 s), indicating that the addition of rGO nanosheets can effectively shorten the induction time and promote the hydration of slag-based geopolymers. Due to the similar characteristics of rGO and GO nanosheets [11], the assumptions proposed for the hydration process of GO-reinforced cement may also be used to explain that of rGO-reinforced geopolymers. First, graphene-based nanosheets are excellent water-sorbents owing to the super-high specified surface area [25]. Therefore, the addition of rGO nanosheets can relatively increase the concentration of OH^−^ ions produced from the decomposition of alkali activators. According to Sun et al. [28], a greater number of OH^−^ ions can accelerate the dissolution of the slag. Second, rGO nanosheets can provide many nucleation sites for the formation of C-S-H and C-A-S-H. As a result, the effect of the delayed nucleation stage is diminished. Finally, the reactions of Ca^2+^ ions and oxygen-containing groups on the rGO surface can decrease the Ca concentration and promote the dissolution of the slag [34]. Consequently, the addition of rGO nanosheets can shorten the induction period, thus promoting the hydration of the slag. The values of the second peak for M0, R1, R2, R3, and R4 are 3.3, 3.6, 3.7, 3.8, and 3.8 mW/g, respectively. This indicates that the hydration degree of the samples increases with the increase of the reduction degree of the rGO nanosheets. In addition, Figure 6 shows the cumulative heat values for M0, R1, R2, R3, and R4. At 150,000 s, the total exothermic heat values for M0, R1, R2, R3, and R4 are 130, 135, 140, 140, and 139 J/g, respectively. This is in line with the results shown in Figure 5b, indicating that the addition of rGO nanosheets increases the hydration degree of slag-based geopolymers. In particular, the value of R4 is lower than that of R3, attributed to the significant structural defects in the rGO nanosheets used in R4.

### 3.3. Chemical Compositions

#### 3.3.1. XRD Analysis

**Figure 7:**
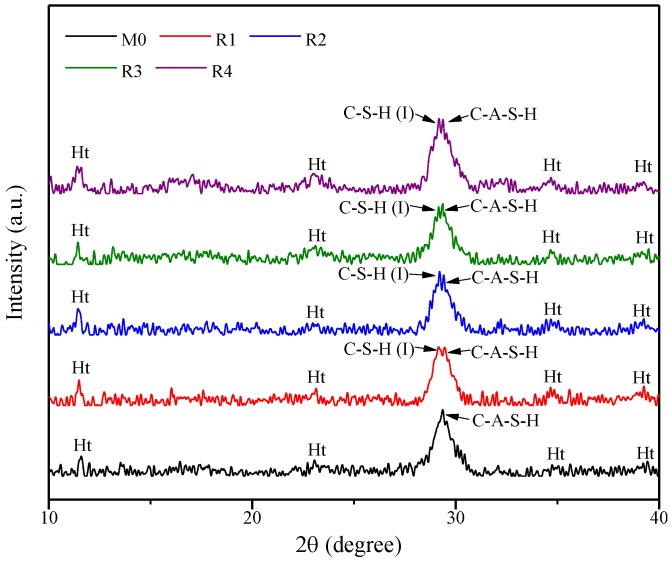
XRD patterns of M0, R1, R2, R3, and R4 after steam curing for 48 h. Ht—hydrotalcite.

To identify the chemical compositions of the geopolymers after rGO addition, the XRD patterns of M0, R1, R2, R3, and R4 after steam curing for 48 h are determined, as shown in Figure 7. The results show that the rGO-reinforced geopolymers have three crystalline hydration products in the range from 10° to 40°. First, the diffusive peak at 2θ = 29.07° is associated with C-A-S-H, which is regarded as C-S-H wherein Al^3+^ is located within its silicate chains, more especially at the tetrahedral bridging sites [35]. Shi et al. [21] pointed out that this peak could be attributed to the generation of tobermorite like C-A-S-H phase (PDF #00-033-0306), while other studies [36,37,38] reported that the C-A-S-H phase can be detected directly owing to its semi-crystalline characteristics [36]. Second, hydrotalcite (Mg_6_Al_2_(CO_3_)(OH)_16_·4H_2_O, PDF #00-014-0191), a type of hydration product penetrating octahedral aluminum, can be identified at peaks of approximately 11.6, 23.3, 34.9, and 39.8°. In particular, hydrotalcite has a layer structure similar to brucite, where the interlayer region contains ${\mathrm{CO}}_{3}^{2-}$ ions and water molecules [39]. Third, the peak on the left side of the C-A-S-H phase can be attributed to the poorly crystalline C-S-H(I) (C-S-H with low Ca/Si ratio, PDF #00-034-0002) [21,40]. The poorly crystalline C-S-H(I) is more ordered than the C-S-H phase [40]. According to Xu et al. [34], the C-S-H(I) phase features a high content of Si and therefore can present as a Si-rich hydrate. Finally, katoite (C_3_AH_6_, PDF #01-011-1713) and strätlingite (PDF #00-014-0631) are not found in the studied samples, although they were detected in other studies.

As shown in Figure 7, M0 contains two main hydration products: hydrotalcite and C-A-S-H. Although Zuo et al. [40] indicated that C-S-H(I) can be detected in alkali-activated slag materials, C-S-H(I) was not found in M0 in this study. In contrast, the C-S-H(I) phase was identified in all the rGO-containing samples, indicating that rGO-reinforced geopolymers prefer to generate C-A-S-H and C-S-H(I) phases simultaneously. This can be attributed to two distinct zones in the rGO–geopolymer pastes: A zone beyond rGO nanosheets (zone 1) and another zone around the rGO nanosheets (zone 2). In zone 1, rGO–geopolymer pastes share the same characteristics with M0, where the C-A-S-H phase is the main hydration product formed. In zone 2, the rGO–geopolymer pastes exhibit some complicated features due to the addition of rGO nanosheets. Since Al element in high alkaline solutions generally exists in the form of electronegative tetrahedron such as ${\mathrm{Al}(\mathrm{OH})}_{4}^{-}$, rGO nanosheets with negative charges would repulse Al-containing phases [34]. This indicates that the Al element around the rGO nanosheets has a relatively low concentration. Moreover, a Si-rich environment can be found around rGO nanosheets owing to the absorption of rGO nanosheets toward Ca^2+^ ions. Therefore, due to the high concentration of Si and the low concentration of Al, the vicinity of rGO nanosheets can form the C-S-H(I) phase rather than C-S-H and C-A-S-H phases. In addition, Figure 7 shows that there are no variations in the chemical compositions between R1, R2, R3, and R4, indicating that the chemical compositions of rGO-reinforced geopolymers are unaffected by the reduction degree of rGO nanosheets.

#### 3.3.2. NMR analysis

**Figure 8:**
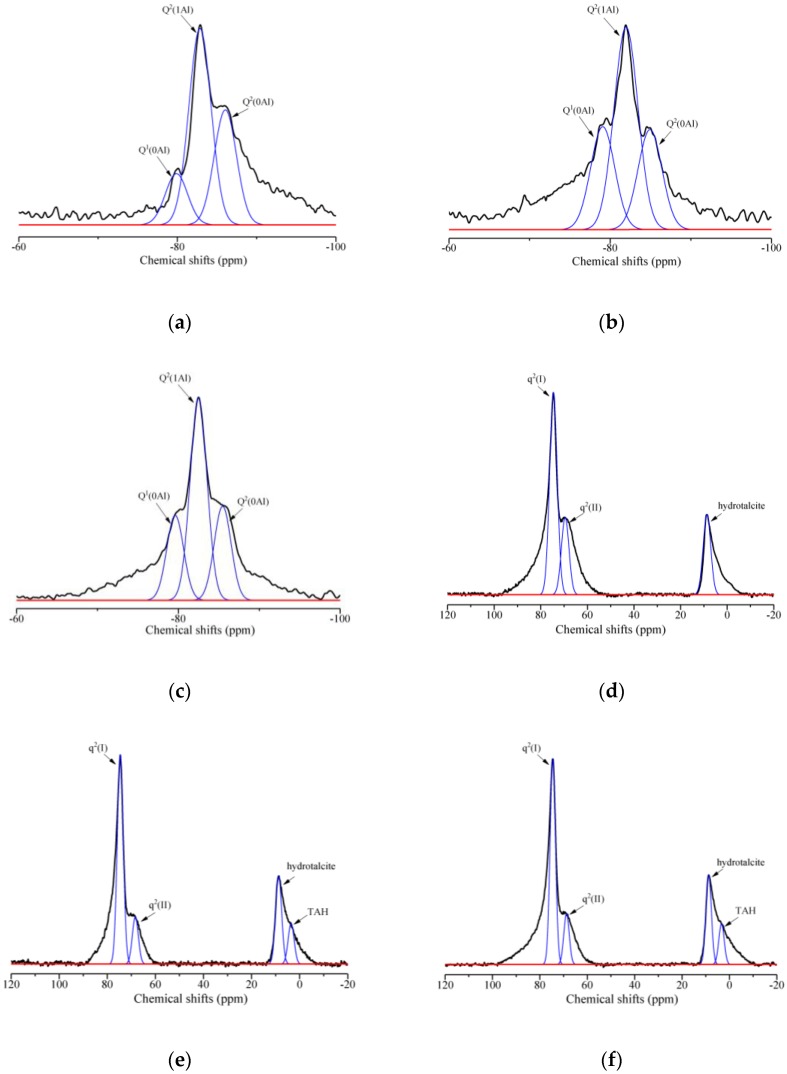
^29^Si magic angle spinning (MAS) nuclear magnetic resonance (NMR) spectra for (**a**) M0, (**b**) R3, (**c**) R4, and ^27^Al MAS NMR spectra for (**d**) M0, (**e**) R3, (**f**) R4.

In addition to the XRD analysis, the chemical structure of the hydration products can also provide important information to explain the effects of rGO addition and reduction degree on the chemical compositions of geopolymers. Figure 8 shows the ^29^Si and ^27^Al MAS NMR spectra for M0, R3, and R4. Since the reduction degree of the rGO nanosheets has no distinct effect on the hydration products of geopolymers (see Section 3.3.1), the NMR analysis for M0, R3, and R4 is sufficient. The MAS NMR spectra of M0, R3, and R4 were semi-quantitatively deconvoluted using a Gaussian function. Table 3 lists the peak assignment and relative integrated area of each peak obtained from the deconvolution.

In the ^29^Si MAS NMR spectra shown in Figure 8a–c, Q*^n^*(*m*Al) notation (*n* = 0–2, *m* = 0–*n*) is used to describe the chemical conditions of Si nuclei, where *n* indicates the number of adjacent Si connected directly to one tetrahedral Si, and *m* represents the number of Al substitutions to the adjacent Si. Here, Al can replace Si in a disorderly manner since the particle radius of Si is similar to that of Al in the silicate structure [32]. As shown in the figures, the peak at around −79.0 ppm can be attributed to the Q^1^(0Al) sites, which represent the end-of-chain silicate tetrahedrons of the C-(A)-S-H (C-S-H or C-A-S-H) gels [41]. Compared with M0, R3 and R4 exhibit an obvious increment in the intensity of Q^1^(0Al) sites due to the repellency of the rGO nanosheets to Al species. This indicates more silicate tetrahedrals in the end of chain of C-(A)-S-H gels in rGO-reinforced geopolymers having no Al substitutions. Two types of Q^2^ groups are also detected in the figures: Q^2^(1Al) sites located at approximately −82.0 ppm and Q^2^(0Al) sites at approximately −85.0 ppm. The Q^2^ sites are assigned to the middle-of-chain silicates of the C-(A)-S-H gels [42,43]. It can be seen that the addition of rGO nanosheets has little effect on the intensity of the Q^2^ sites in the geopolymers. This suggests that the presence of the C-S-H(I) phase in the rGO-reinforced geopolymers (see Section 3.3.1) is mainly due to the fact that the rGO nanosheets can restrain the substitution of Al on the end-of-chain silicates of C-(A)-S-H gels rather than the middle-of-chain silicates. In addition, the peak at approximately −75.0 ppm generally corresponds to the Q^0^ sites, attributed to the isolated silica tetrahedrons from unhydrated slag particles [44]. In particular, the Q^0^ sites were not found in M0, R3, and R4, indicating that the alkali activation of the slag is almost complete under steam curing at 80 °C for 48 h.

Table 3 shows that the integrated area percentage of Q^1^(0Al) increases from 4.44% to 11.14% and 10.02% due to the addition of rGO nanosheets, whereas that of Q^2^(1Al) decreases from 55.47% to 45.80% and 47.76%. Moreover, a slightly upward trend can be found in Q^2^(0Al). This confirms that the rGO nanosheets can restrain the substitution of Al on the end-of-chain silicates of C-(A)-S-H gels. In addition, a comparison between R3 and R4 shows that the rGO nanosheets from alkali reduction under 80 °C have significant structural defects, thus restricting its effect on Al substitution in hydration products of geopolymers.

In theory, three distinct Al environments, namely Al(IV), Al(V), and Al(VI), can be observed in the ^27^Al MAS NMR spectra of the geopolymer pastes. The peaks at approximately 74.0 ppm and 68.0 ppm, q^2^(I) and q^2^(II), are assigned to the tetrahedral Al(IV) incorporated in the C-A-S-H gels under bridging conditions [45]; the peak in the range from 30.0 to 40.0 ppm is attributed to the presence of Al(V); the peak with lower intensity in the range from 0 to 20.0 ppm is generally considered octahedral Al(VI) in AFm and/or hydrotalcite phases [46]. As shown in Figure 8, the peak assigned to Al(V) was not found in all the samples, and hydrotalcite was identified given the absence of the AFm phase in the XRD patterns. Compared with M0, the intensities of the q^2^(II) sites in R3 and R4 remarkably decrease due to the addition of rGO nanosheets; this is consistent with the ^29^Si MAS NMR analysis results, which show that the addition of rGO nanosheets decreases the intensity of the Q^2^(1Al) sites. This indicates that the addition of rGO nanosheets can hinder the substitution of Al in the silicate chain of C-A-S-H gels, thus promoting the formation of C-S-H(I) phases. In addition, R3 and R4 have a new hydration product, namely third aluminate hydrate (TAH) [47]. This can be attributed to the increase of the number of Al phases in other hydration reactions due to the repellency of rGO nanosheets with the negative charges to Al species.

### 3.4. Flexural Strength

**Figure 9:**
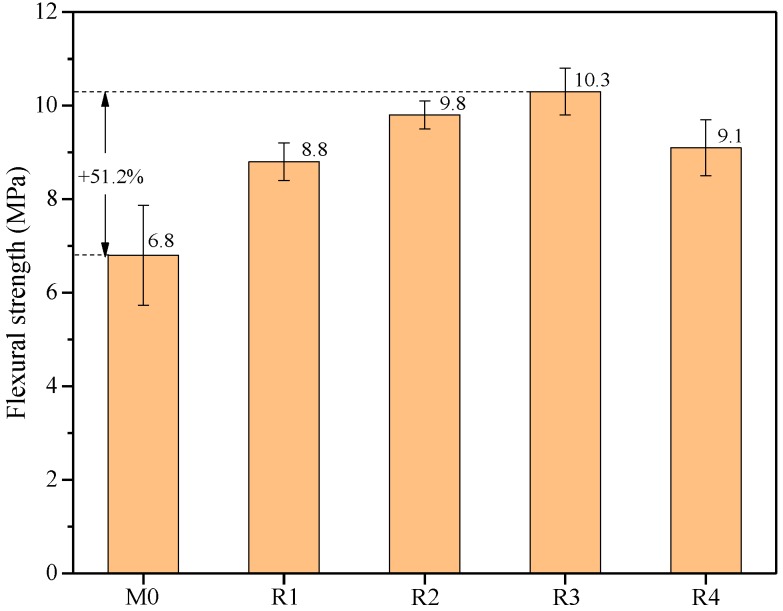
Flexural strengths of M0, R1, R2, R3, and R4 after steam curing at 48 h.

Although geopolymers have a few distinct advantages over OPC, including greater environmental benefits and better durability, their low flexural strength hinders their engineering application. Thus, rGO nanosheets are used to enhance the flexural strength of geopolymers. Figure 9 shows the effects of rGO addition and reduction degree on the flexural strength of the geopolymer mortars after steam curing at 48 h. When the treatment temperature is within 60 °C, the flexural strength of the samples gradually increases from 6.8 to 10.3 MPa. An obvious increment of 29.4% on the flexural strength is observed in R1, whereas R2 and R3 exhibit a small increment in the flexural strength. In particular, R3 exhibits the maximum flexural strength, which is 51.2% higher than that of M0. In addition, the flexural strength of the samples decreases with the increase of the treatment temperature from 60 to 80 °C. This indicates that the significant structural defects in rGO nanosheets under alkali reduction at 80 °C can hinder the reinforcing effect.

Although rGO nanosheets have been proven to improve the flexural strength of geopolymers, the related mechanism is unclear. Thus, a synergistic analysis of hydration process and chemical composition is performed. Owing to the super-high specific surface area, rGO nanosheets can absorb a certain amount of free water and increase the relative concentration of OH^−^ ions, thus promoting the dissolution of the slag (see Section 3.2). Similarly, the superior specific surface area can provide many nucleation sites for the formation of C-S-H and C-A-S-H gels. As a result, rGO addition can improve the hydration degree of the slag. Generally, a higher hydration degree can result in the formation of a denser microstructure [38]. On the other hand, due to the repellency of rGO nanosheets with negative charges to Al species, the substitution of Al on the end-of-chain silicates of C-(A)-S-H gels is restrained (see Section 3.3.2). Meanwhile, due to the absorption of rGO nanosheets to Ca species, a region with a high concentration of Si and a low concentration of Al is formed around the rGO nanosheets, which results in the formation of C-S-H(I) gels. Moreover, C-A-S-H gels can be generated beyond the rGO nanosheets (see Section 3.3.1). According to Richardson et al. [48], different types of C-(A)-S-H gels possessing various morphologies and chemical compositions exhibit different contributions to the mechanical properties of alkali activated composites. Yip et al. [49] also reported that the simultaneous formation of multiple gels helps to bridge the gaps among different phases and unhydrated particles, leading to the superior mechanical strengths of slag-based geopolymers. Consequently, an accelerated hydration of the slag and the simultaneous formation of C-S-H(I) and C-A-S-H gels contribute to the improvement in the flexural strength of rGO-reinforced geopolymers.

## 4. Conclusions

In this study, rGO nanosheets with different reduction degrees were successfully prepared to investigate the effects of the reduction degree on the hydration process, chemical composition, and flexural strength of geopolymers. In addition, the reinforcing mechanism of rGO nanosheets on the flexural strength of geopolymers was illustrated using a synergistic analysis of the hydration process and chemical composition. The following conclusions can be drawn from the test results:

1. With the addition of rGO nanosheets, the hydration degree of the samples significantly increases, since rGO nanosheets increase the relative concentration of OH^−^ ions and provide many nucleation sites for the formation of C-S-H and C-A-S-H gels. When the reduction temperature is 60 °C, the hydration degree of rGO-reinforced geopolymers is the highest with a cumulative heat of approximately 140 J/g at 150,000 s.
2. With the addition of rGO nanosheets, the samples prefer to generate C-S-H(I) and C-A-S-H phases simultaneously due to the repellency of rGO nanosheets with negative changes toward Al species. In addition, the reduction degree has no obvious effect on the chemical composition of rGO-reinforced geopolymers.
3. With the addition of rGO nanosheets, the integrated area percentage of Q^1^(0Al) increases from 4.44% to 11.14%, whereas that of Q^2^(0Al) has no obvious change, indicating that rGO nanosheets largely restrain the substitution of Al on the end-of-chain silicates of C-(A)-S-H gels rather than the middle-of-chain silicates.
4. With the addition of rGO nanosheets, the flexural strength of the samples remarkably increases. The flexural strength of R3 is 51.2% higher than that of M0, owing to the promotion of slag hydration and the simultaneous formation of C-S-H(I) and C-A-S-H gels. In addition, the flexural strength of R4 is lower than that of R3 due to the significant structural defects in rGO nanosheets under alkali reduction at 80 °C.

## Figures and Tables

**Table 1:** Chemical composition and physical properties of slag.

Component (wt %)		Slag
Chemical compositions	CaO	39.08
	SiO_2_	35.12
	Al_2_O_3_	14.20
	Fe_2_O_3_	0.62
	MgO	8.47
	TiO_2_	0.71
	MnO	0.69
	Na_2_O	0.50
	K_2_O	0.48
	Loss on ignition (%)	0.10
Physical properties	Bulk density (g/cm^3^)	1.20
	Density (g/cm^3^)	2.90
	Specific surface area (m^2^/kg)	430.00

**Table 2:** Properties of graphite oxide.

Appearance	Solid Content (mass %)	pH	Carbon (mass %)	Oxygen (mass %)	Hydrogen (mass %)
Black paste	54.22	2.40	46.03	46.68	2.84

**Table 3:** Peak assignment and integrated area percentage of deconvoluted ^29^Si MAS NMR spectra peaks.

Chemical Shifts (±1 ppm)	Site Type	Sample ID		
		M0	R3	R4
−85	Q^2^(0Al)	40.09	43.06	42.22
−82	Q^2^(1Al)	55.47	45.80	47.76
−79	Q^1^(0Al)	4.44	11.14	10.02
